# Comparing a single-stage geocoding method to a multi-stage geocoding method: how much and where do they disagree?

**DOI:** 10.1186/1476-072X-6-12

**Published:** 2007-03-16

**Authors:** Gina S Lovasi, Jeremy C Weiss, Richard Hoskins, Eric A Whitsel, Kenneth Rice, Craig F Erickson, Bruce M Psaty

**Affiliations:** 1 Columbia University, Institute of Social and Economic Research and Policy, New York, NY, USA; 2 University of Washington, Cardiovascular Health Research Unit, Seattle, WA, USA; 3 Washington State Department of Health, Olympia, WA, USA; 4 University of North Carolina, Departments of Epidemiology and Medicine, Chapel Hill, NC, USA; 5 University of Washington, Department of Biostatistics, Seattle, WA, USA; 6 University of Washington, Departments of Epidemiology, Medicine, and Health Services, Seattle, WA, USA

## Abstract

**Background:**

Geocoding methods vary among spatial epidemiology studies. Errors in the geocoding process and differential match rates may reduce study validity. We compared two geocoding methods using 8,157 Washington State addresses. The multi-stage geocoding method implemented by the state health department used a sequence of local and national reference files. The single-stage method used a single national reference file. For each address geocoded by both methods, we measured the distance between the locations assigned by each method. Area-level characteristics were collected from census data, and modeled as predictors of the discordance between geocoded address coordinates.

**Results:**

The multi-stage method had a higher match rate than the single-stage method: 99% versus 95%. Of 7,686 addresses were geocoded by both methods, 96% were geocoded to the same census tract by both methods and 98% were geocoded to locations within 1 km of each other by the two methods. The distance between geocoded coordinates for the same address was higher in sparsely populated and low poverty areas, and counties with local reference files.

**Conclusion:**

The multi-stage geocoding method had a higher match rate than the single-stage method. An examination of differences in the location assigned to the same address suggested that study results may be most sensitive to the choice of geocoding method in sparsely populated or low-poverty areas.

## Background

Spatial epidemiology studies often begin with address geocoding, allowing residences, facilities, or other structures to be geographically located and placed in the context of their surroundings. Errors in the geocoding process lead to incorrect location assignment and misclassification of the corresponding data [1]. Often, however, there is a trade off between minimizing positional error and maximizing the geocoding match rate (percentage of addresses located) [2]. That is, a process or setting may have a higher match rate at the expense of placing the address far from its location, or a higher confidence in the assigned locations at the expense of the match rate. Krieger [3] has encouraged public health researchers to evaluate and report on their geocoding methods, and a number of recent papers have done so [2-10]. In this paper, we compared two geocoding methods: an automated, single-stage method and a multi-stage method used by the Washington State Department of Health (WA DOH).

MacDorman [11] found that 21 (43%) of the 49 surveyed state vital statistics departments reported some automated geocoding of address data from vital records. Software tools used varied widely, including in-house software, Matchmaker, Dynamap, Code 1, ArcInfo/ArcView, Finalist/Final Focus, and others. Also, several states subcontracted their geocoding to outside agencies. The WA DOH has an established multi-stage geocoding protocol (described in detail on their website [16]), which is made available to local health departments and spatially-oriented public health projects throughout the state. The WA DOH uses proprietary address standardization software, local reference data where available, and Topologically Integrated Geographic Encoding and Referencing system (TIGER)-based street files from up to four different years [12]. This method required investment in software, proprietary geographic reference data, and programming time. We evaluated how much is gained through a multi-stage process like that of WA DOH, compared to a simpler, single-stage process, by looking at how and where these two methods disagreed.

Using a sample of 8,157 Washington State addresses, we compared the WA DOH multi-stage geocoding method to a single-stage geocoding method based on a single street reference file. We expected to find a higher geocoding match rate with the WA DOH multi-stage process. For addresses geocoded by both methods, we measured the distance between multi-stage and single-stage geocoded coordinates for the same address; we use this "discrepancy-distance" to quantify disagreement between the two methods. We expected that the multi-stage and single-stage geocoded address coordinates would be more similar, and discrepancy-distances smaller, in more densely populated areas and areas where national street files were used as a reference for both geocoding methods. Further, we hypothesized that the two geocoding methods would disagree less, as indicated by smaller discrepancy-distances, in low poverty areas.

## Results

Of the 8,157 Washington State addresses, we were able to geocode 8,098 (99%) by at least one method and 7,686 (95%) addresses by both methods. The multi-stage geocoding process matched 8,058 (99%) of the addresses, and the single-stage geocoding method matched 7,726 (95%).

While we included addresses in each of the 39 counties in Washington State, more of the geocoded addresses were in the densely populated counties. According to Census data from the year 2000, Washington State had an overall density of 34 residents per square kilometer; 68% of our geocoded addresses were in the 8 counties with densities higher than 34 residents per square kilometer. In the state, 10% of the population was below the federal poverty line; 51% of our geocoded addresses were in counties with less than 10% poverty.

While local reference data (tax parcels or street files from local government agencies) have been recommended for greater geocoding accuracy [9,13,14], these data are not uniformly available. Of all Washington State residents in the year 2000, 61% lived in counties with local street data and 68% lived in counties with parcel data; 59% and 63% of the geocoded addresses in our study were in counties with local street data and parcel data, respectively.

Compared to addresses geocoded by both methods, those geocoded by only the multi-stage method were less likely to be in counties with parcel data (Table 1); addresses geocoded only by the single-stage method were less likely to be in counties with local street data and tended to be in sparsely populated areas. Addresses geocoded by only one method, rather than both methods, also tended to be in areas with higher poverty rates.

**Table 1:** Area characteristics for geocoded addresses

**Geocoded by:**	**Both methods**	**Multi-stage method only**	**Single-stage method only**
	N = 7686	N = 372	N = 40
Parcel data available, %	63%	48%	55%
Local street data available, %	59%	66%	35%
Density, median, population/km^2^			
County	111	91	21
Census tract	980	1177	60
Census block group	1183	1280	133
Percent poverty, median			
County	9%	11%	14%
Census tract	9%	12%	10%
Census block group	8%	12%	10%

Area characteristics were based on the multi-stage geocoded address coordinates when available (N = 8,058) and the single-stage geocoded address coordinates otherwise (N = 40).

For those addresses matched by both methods, 96% (7,374) were geocoded to the same census tract by each method; of addresses in the same census tract, 93% (6,859) were geocoded to the same census block group by both methods. The density and percent poverty based on the two geocoding results generally agreed as well: for density, intraclass correlation coefficients were 0.97, and 0.93 at the census tract and census block group levels, respectively; for percent poverty, intraclass correlation coefficients were 0.97 and 0.89 at the census tract, and census block group levels.

**Figure 1:**
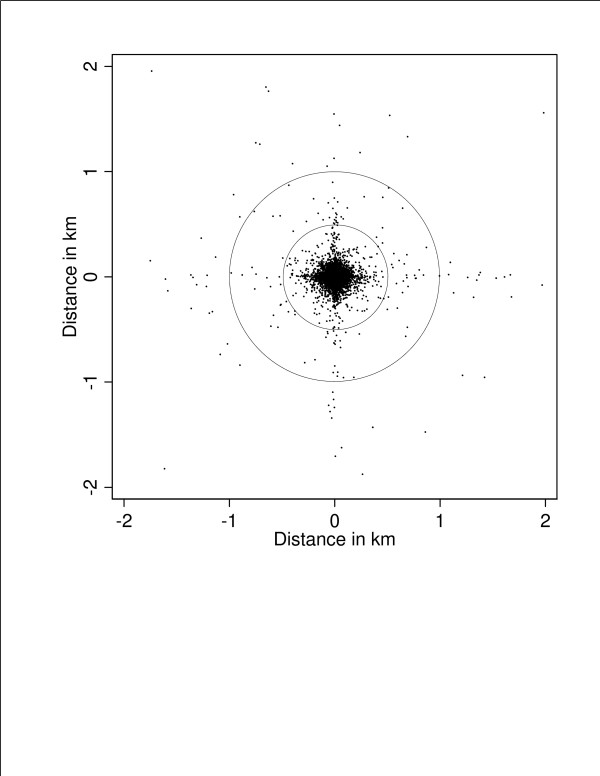
**Distance and directional bias between geocoded address coordinates for multi-stage and single-stage geocoding methods**: This figure shows one dot for each address geocoded by both methods, with reference circles at 0.5 and 1.0 km. The multi-stage geocoded address coordinates was centered as a reference, and the dots used to show the relative position of the single-stage address coordinates for the same address. Dots close to the middle (0,0) represent small discrepancy-distances and high concordance between the two methods. Dots directly above the center had single-stage geocoded address coordinates further north than their multi-stage coordinates. Dots randomly scattered in all directions would indicate no directional bias, whereas an off-center cluster of dots would indicate systematic bias between the two methods. Addresses with discrepancy distances greater than 2 km were not included in this figure (N = 78).

**Figure 2:**
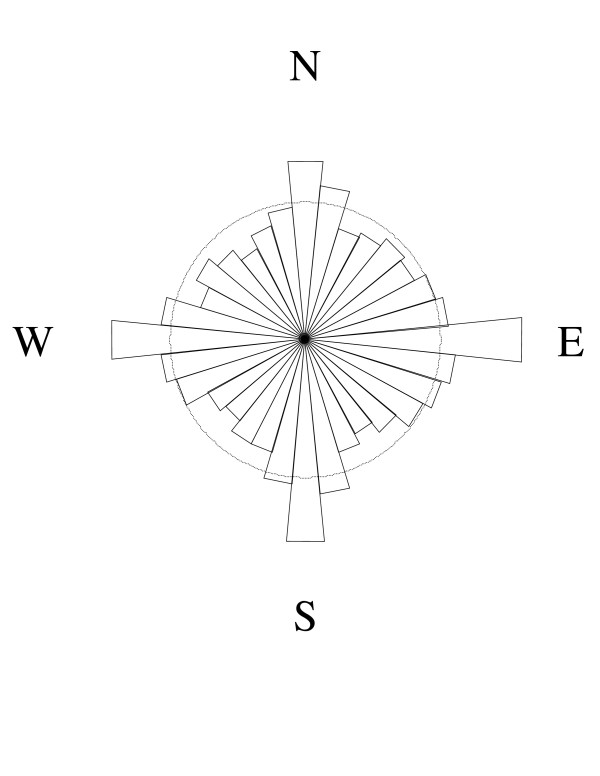
**Directional bias between geocoding methods**: This figure shows an angular histogram with radial lengths proportional to frequencies of shifts in each direction between the single-stage and multi-stage geocoded address coordinates. A light circle is drawn at the mean frequency for reference.

Although the locations assigned by the two geocoding methods differed, these differences did not take the form of a systematic shift in one direction (Figure 1). A cloud of dots shifted off-center relative to the reference circles would have suggested systematic bias. While there was no single direction bias, however, we did observe clustering along the north-south and east-west axes (Figure 2). A *post-hoc *examination of subgroups suggested that the clustering along axes was most pronounced for counties where the multi-stage method used local roads for geocoding. We confirmed this "plus" pattern in a repeat analysis using identical offsets for both geocoding methods.

**Figure 3:**
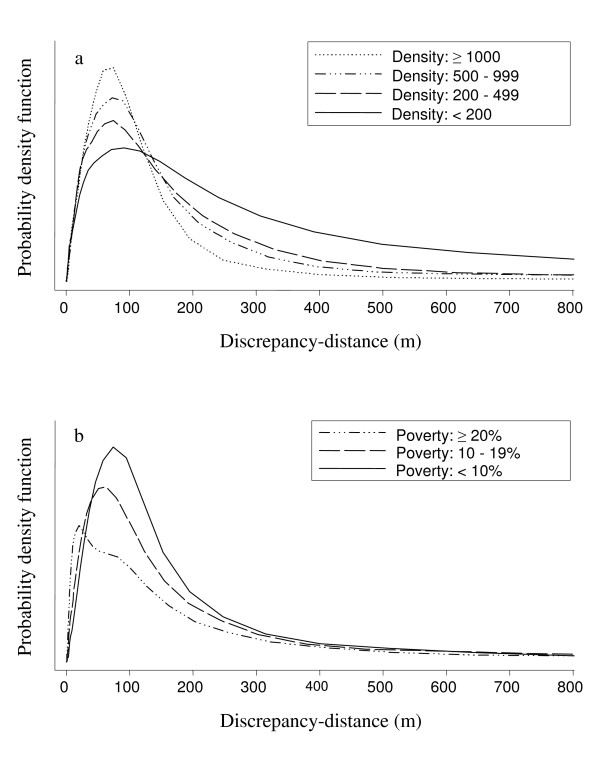
**Discrepancy-distance distributions by category of (a) density and (b) poverty**: This figure shows a smoothed kernel density (similar to a histogram) for discrepancy-distances by category. Large discrepancy-distances indicate disagreement between the single-stage and multi-stage geocoding methods. Density is categorized using the number of residents per square kilometer in the census tract. Poverty is categorized using the percent of residents below the poverty line in the census tract.

The median discrepancy-distance between locations assigned by the two methods was 54 meters, and the 90^th ^and 95^th ^percentiles were 180 and 296 meters (Table 2). The discrepancy-distance was less than 1 km for 98% of the addresses. Discrepancy-distances were largest in low-density census tracts (Figure 3a). The associations of discrepancy-distance with census tract poverty and multi-stage data source were not consistent. Medians and 90^th ^percentiles suggested that discrepancy-distances were larger in areas with less poverty, but the means and proportion larger than 1 km suggested the largest discrepancy distances were for areas with between 10 and 20% poverty (Table 2, Figure 3b). Areas where the WA DOH multi-stage process used local parcel data had a higher median discrepancy-distance, but fewer discrepancy-distances were greater than 1 km in these counties.

**Table 2:** Distance between locations for the same address assigned by two geocoding methods, by area characteristics

	**Discrepancy-distance (m)**								
					**Percentiles**				
						
		**N**	**Arith. mean (95% CI)**	**Geo. mean (95% CI)**	**50th**	**90th**	**95th**	**99th**	**Percent > 1 km apart**

**Overall**		7,686	160 (140, 179)	49 (48, 51)	54	180	296	2218	1.8%
									
Census Tract Poverty*	≥ 20%	1,027	156 (77, 234)	28 (25, 30)	26	144	258	1370	1.4%
	10 to 19%	2,248	169 (135, 203)	46 (44, 49)	48	183	310	3052	2.2%
	< 10 %	4,411	156 (132, 180)	59 (57, 61)	62	187	300	1873	1.7%
									
Census Tract Density* (population/km^2^**)**	≥ 1000	3,770	106 (83, 129)	44 (43, 46)	50	134	174	717	0.8%
	500 – 999	1,504	141 (108, 174)	50 (47, 53)	55	175	269	1970	1.6%
	200 – 499	931	213 (140, 286)	49 (45, 54)	52	202	333	5593	2.5%
	< 200	1,481	281 (218, 345)	66 (61, 71)	70	401	779	5548	4.1%
									
Most common reference for multi-stage method*	Local Parcels	4,644	152 (128, 176)	61 (59, 62)	63	172	258	1704	1.5%
	Local Roads	1,075	136 (107, 165)	52 (48, 56)	53	193	333	1667	2.0%
	TIGER-based	1,967	191 (140, 242)	30 (28, 32)	25	211	414	4028	2.5%

Discrepancy-distance indicates distance between multi-stage and single-stage geocoded address coordinates for the same address; p50, p90, p95, and p99 indicate the 50^th^, 90^th^, 95^th^, and 99^th ^percentiles; TIGER indicates Topologically Integrated Geographic Encoding and Referencing system line files, this includes NAVTEQ and Dynamap reference files

* Indicates significance (p < 0.05) for a comparison of discrepancy-distances across subgroups using a linear regression model

In multiple regression, accounting for poverty, density, and multi-stage reference file simultaneously, we found larger discrepancy-distances for (1) areas with lower density, (2) areas with a lower percent poverty, and (3) counties with local parcel data or local roads (Table 3). As density doubled, the median discrepancy-distance decreased by approximately 10%, indicating closer agreement in densely populated areas for the two geocoding methods. As poverty doubled, the median discrepancy-distance decreased by approximately 13%, indicating closer agreement between geocoding methods in high poverty areas. Where the multi-stage geocoding method used mainly TIGER-based street files, median discrepancy-distance was halved compared to counties where local parcel files were the most common reference. When corresponding models were run at the county and census block group levels, estimates for density and poverty remained significant and estimates were in the same direction.

**Table 3:** Multi-variable regression model of discrepancy-distance

		Ratio of discrepancy-distance medians* (95% CI)
**Poverty in census tract**	Two-fold increase	0.87 (0.85 – 0.91)
**Density of census tract**	Two-fold increase	0.90 (0.88 – 0.91)
**Multi-stage reference file**	Local Parcels	1.00 (reference)
	Local Roads	0.89 (0.82–0.96)
	TIGER-based	0.47 (0.44–0.51)

Discrepancy-distance indicates distance between multi-stage and single-stage coordinates for the same address; TIGER indicates Topologically Integrated Geographic Encoding and Referencing system line files, this includes NAVTEQ and Dynamap reference files

* Ratios lower than one indicate that a category or characteristic was associated with a smaller discrepancy-distance (closer agreement between the two geocoding methods)

In stratified analyses, the effects of poverty and density were in the same direction, but were most pronounced where both geocoding methods used TIGER-based street files: median ratio was 0.81 (95% CI: 0.75 – 0.86) for doubling poverty in this subgroup (likelihood ratio test p for interaction: < 0.001) for a doubling of density and the ratio was 0.86 (95% CI: 0.83 – 0.88).

## Discussion

We found that a multi-stage geocoding method implemented by the WA DOH achieved a match rate 4% higher than that achieved by a single-stage method. Most addresses were matched by both methods, but they were not geocoded to exactly the same coordinates by each method: 10% of addresses were assigned locations at least 180 meters apart by the multi-stage and single-stage methods, and 2% of addresses were assigned locations at least one kilometer apart. Locations assigned by the two methods were closer together in high density and high poverty areas, and in areas where reference data sources were most similar for the two methods. The results for area-level poverty, which were contrary to our hypothesis, were not explained by density or availability of local reference files. The associations of area-level poverty and density with discrepancy-distance were strongest where the two methods used similar reference files.

Previous studies have evaluated different single-stage geocoding methods [9] or geocoding vendors [2-4], automated versus interactive geocoding methods [5], or compared a single-stage geocoding method with a gold standard [7,8]. Also, McElroy described and recommended the use of a multi-stage geocoding process, despite added costs [10]. Our study contributes to this literature by (1) providing further information on geocoding results of a multi-stage process as compared to a single-stage process, (2) confirming previous findings that geocoding methods may have better agreement in densely populated areas [4,7-9], and (3) suggesting that geocoding methods may also have better agreement in high poverty areas, after controlling for population density. Geocoding discrepancies in low poverty areas could be due to differences in address quality, reference file quality, or other determinants of geocoding error (such as recent redevelopment, street length, or lot size). If this association is confirmed, further research will be needed to distinguish among these possibilities.

Our study investigated whether single-stage geocoded address coordinates were systematically shifted relative to the multi-stage address coordinates. We found that the single-stage coordinates were shifted north-south and east-west relative to the multi-stage coordinates more often than would be expected by chance alone. This may have been due to different assumptions about how addresses are spaced along a street [14], since WA streets are more likely to be oriented in the cardinal directions than would be expected by chance. In Washington State, we estimated that 42 percent of street segments are within five degrees of being oriented directly north-south or east-west (only 11 percent expected by chance). This directional shift finding may be most relevant to areas where urban planners played an active role in establishing N-S and E-W roadway grids.

Since the accuracy of address geocoding depends on address quality, preprocessing, program settings and reference maps [15], further research is needed to understand the effects of each component. While our study and others have controlled for address quality by using the same addresses for both methods, we simultaneously examined differences in preprocessing, geocoding software, and reference maps. A limitation of our study is that we did not discern which elements contributed most to the difference between the two geocoding methods under investigation, and cannot use these data to project differences between other approaches. We considered only one of many possible contrasts between geocoding methods, by comparing one multi-stage process to one single-stage process. Given that these two methods were implemented independently, using different software packages and reference files, this contrast may provide an upper bound for how much geocoding methods in large research studies would be expected to differ for a state-wide administrative data set. Also, we had no gold standard with which to evaluate the relative accuracy of the two geocoding methods; however, a third geocoding method using satellite images (implemented using Google Earth Pro, as described below in the Methods section on *Supplemental geocoding*) agreed more closely with the multi-stage geocoding method. Rather than focusing on comparisons with a gold standard, we evaluated which area characteristics predicted larger discrepancies between two geocoding methods.

Another limitation was that there may have been unmeasured or residual confounding by address or area characteristics in this study, interfering with our ability to assess which characteristics predicted geocoding discrepancies. Finally, the geographic scope and distribution of our study addresses limits the generalizability of this Washington-based study [4]. These data were statewide and may be similar to other health department address data; however, the geocoded addresses were all numbered street addresses with ZIP codes and did not include Post Office boxes.

The importance of the differences between any two methods depends on the context and purpose of geocoding. Both the level of analysis and hypothesized exposure effects will influence the cost of geocoding errors. The available data or confidentiality protections may constrain some researchers to work with data at the zip code or census tract level, or even "jittered" address locations with deliberately introduced error. Some researchers might find our 96% concordance at the census tract level encouraging. Single-stage geocoding using street addresses may be adequate for some research purposes. However, for a study of small-scale environmental exposures, such as radiation, the ability to detect or replicate an association may depend on the geocoding method selected, and even multi-stage geocoding may place addresses far from their actual locations. The importance of relative geocoding precision may also vary across areas. For example, the commonly observed pattern of decreased geocoding accuracy in sparsely populated areas may be of little concern if an exposure, such as air pollution, is less variable across small distances in a rural context. Geocoding match rates which vary among geocoding methods can also affect the power and external validity [6] of spatial epidemiology studies; subjects with unmatched addresses may not be representative and are generally excluded from further analyses.

We refer those choosing a geocoding method for a particular study, research group or health department to previously published reviews and recommendations [1,3,6,14]. Based on our experience, even a group with limited resources and time can incorporate geocoding through a low-cost, single-stage method, like the one described here. This is likely to be adequate when (1) the addresses are relatively free of spelling and formatting errors, as may be the case with billing addresses; (2) the addresses of interest are mainly in high density or high poverty areas; and (3) the exposure of interest varies only gradually with distance. For organizations like the Washington State DOH, initial costs for setting and validating a multi-stage system may facilitate a variety of projects by improving match rates and utilizing local geographic files when available. Another option, not evaluated here, would be using a commercial geocoding vendor [2,4,7].

## Conclusion

The multi-stage geocoding method examined in our study had the advantage of a higher match rate, but without a gold standard with which to gauge the accuracy of the two geocoding methods we could only guess the relative validity of the two methods. Our findings and those of previous studies suggest that the choice of geocoding method may be especially influential in areas with low density or low poverty.

## Methods

### Address data

A sample of addresses throughout Washington State was geocoded using the multi-stage WA DOH method and a single-stage method within a GIS software package. This convenience sample included 8,753 addresses of licensed daycare providers in Washington State, collected from 2003–2005 by the Children's Administration of the Washington State Department of Social and Health Services.

All addresses had an accompanying ZIP code, but two were street intersections and 156 others had no street number. The addresses without street numbers were not geocoded by either method. There were also 475 Post Office boxes and two Mail Stop numbers. After intersections, addresses without a street number, Post Office boxes and Mail Stops were excluded, 8,157 addresses remained in our analyses.

### Multi-stage geocoding

The multi-stage geocoding method used by the WA DOH is documented online [16]. This method began with automated preprocessing: automated address correction, standardization to United States Postal Service (USPS) format, and parsing. Preprocessing was done using Centrus software [17].

After preprocessing, addresses were geocoded in stages by matching them to different reference maps of tax parcels or streets using ArcView 3.2 [18]. At each stage, the remaining unmatched addresses were geocoded using a different reference file. Tax parcel data linking addresses to geographic coordinates were used first, accounting for 45% of the address matches, but were used in only 8 of 39 counties. For those addresses not matched to parcels, the following street reference files were used, in this order: local street reference files (available for 14 counties in Washington State, 21% of address matches); enhanced TIGER-based NAVTEQ GPS Streets (from Navigation Technologies [19], 25% of address matches); and TIGER-based line files (including the Geographic Data Technology Dynamap, provided with ArcView [18]). In addition to year 2000 TIGER-based line files (accounting for 8% of address matches), TIGER-based line files from three other years were used (1998, 1995, 1992); these earlier years were used to match only one percent of the study addresses. Geocoding was attempted for each reference file using strict criteria to identify a match, and these stages were repeated for addresses that had not yet been matched using less stringent match criteria. This geocoding sequence was executed using custom-written program scripts in the language Avenue [18].

To assess data accuracy/availability at the county level, we recorded the type of reference file (local parcel, local street, or TIGER-based street file) most commonly used for the multi-stage geocoding process for each county. We also categorized counties according to whether local parcel and street data, collected from 2000 to 2003, were available to WA DOH at the time of multi-stage geocoding.

### Single-stage geocoding

Single-stage geocoding for this project was done using Maptitude GIS software, version 4.7 [20]. This software was selected as representative of GIS software products that include street files (value-added TIGER files), and because address standardization to USPS format is done as part the geocoding process. Directional prefixes (e.g. the "N" in "N 123 Fourth St") in 68 addresses prevented the single-stage method from finding a match, but 97% of these addresses were matched by the multi-stage method.

We used the default geocoding setting to identify exact or approximate ("normal") street address matches within the provided postal code. In a sensitivity analysis, a very strict match criterion reduced the single-stage match rate to 67 percent, and reduced the proportion of discrepancy-distances above one kilometer to 0.5 percent (mean discrepancy-distance was reduced from 160 meters to 91 meters). Another sensitivity analysis showed that changing the offset from 25 to 30 feet in order to match the offset of the multi-stage method did not change any of the results substantially.

### Supplemental geocoding

In order to explore the accuracy of the multi-stage and single-stage geocoding methods, we used Google Earth Pro (version 3.0, released November 17, 2005) to geocode a sample of the study addresses. This sample included all of the addresses matched by only one method and a random sample of 1000 addresses matched by both the single-stage and multi-stage geocoding methods. While not a perfect gold standard, Google Earth Pro incorporates information from satellite images during the relevant time period, 2003 to 2005. Supplemental geocoding results were similar to both multi-stage and single-stage geocoding results for addresses matched by both of these methods (Table 4). For the small number of addresses originally geocoded by the single-stage method but not the multi-stage method, supplemental geocoding did not correspond as closely to the single-stage result.

**Table 4:** Supplemental geocoding results using satellite images

**Original geocoding result**	**Single-stage only**	**Multi-stage only**	**Both methods**
**Number selected**	40	372	1000 (a random sample)
**Number (%) matched by Google Earth Pro**	24 (60)	233 (63)	962 (96)
**Distance to single-stage geocoded location, meters**			
10^th ^percentile	17	.	10
25^th ^percentile	57	.	19
50^th ^percentile (median)	172	.	38
75^th ^percentile	871	.	83
90^th ^percentile	3402	.	162
Proportion > 1000, %	25.0	.	1.5
**Distance to multi-stage geocoded location, meters**			
10^th ^percentile	.	6	6
25^th ^percentile	.	8	11
50^th ^percentile (median)	.	30	32
75^th ^percentile	.	67	65
90^th ^percentile	.	153	125
Proportion > 1000, %	.	3.9	1.8

### Area characteristics

For both the multi-stage and single-stage address coordinates we collected information on characteristics of the surrounding county, census tract, and census block group using a point-in-polygon process. A point-in-polygon process was used, despite the limitations of this method [14], for consistency across geocoding reference files. Percent of poverty was selected as a measure of neighborhood socioeconomic status [21]. Density (population/area) and poverty (percent of individuals below the federal poverty line) were based on the 2000 Census. These data are included with the Maptitude software for the county and census tract levels [20], and were obtained for census block groups through the Washington State Geospatial Data Archive [22].

Intra-class correlation coefficients for census tract density (0.24, 95% CI: 0.08 to 0.41) and poverty (0.31, 95% CI: 12 to 51) by county indicated that census tracts in the same county tend to be similar. Likewise, intra-class correlation coefficients for census block group density (0.69, 95% CI: 0.66 to 0.71) and poverty (0.68, 95% CI: 0.65 to 0.71) by census tract indicated that census block groups in the same census tract tend to be similar. The correlation between poverty and density in our Washington State sample varied by level of measurement: -0.66, 0.16 and 0.21 for county, census tract, and census block group levels, respectively.

Addresses were primarily described using the characteristics of areas surrounding the multi-stage address coordinates. For the addresses geocoded by both methods, we examined concordance by area and area characteristics. For the few addresses geocoded only by the single-stage geocoding method (N = 42), we used areas surrounding the single-stage coordinates.

### Location comparisons

Distance between the multi-stage and single-stage geocoded address coordinates (discrepancy-distance) was used to describe how these two geocoding methods would differ for spatial epidemiology studies. To calculate distance from longitudes and latitudes we used the Haversine formula [23]. A greater discrepancy-distance would indicate more difference between the results of the two methods. The degree of difference may result from lower precision for one or both methods, or from a shift between the reference files used by the two methods. The distribution of discrepancy-distances across categories was shown using smoothed kernel density plots.

We used a bulls-eye plot [4,8] to show the directional bias of single-stage geocoded address coordinates with respect to the corresponding multi-stage coordinates. Each dot on this plot represents one address that was geocoded by both methods. The multi-stage geocoded address coordinates were placed at the origin for each address, and the relative position of the single-stage coordinates was shown as a dot. Dots close to the center (0,0) had small discrepancy-distances. A dot directly above the center had a single-stage geocoded address coordinates further north than the corresponding multi-stage coordinates, and a discrepancy-distance equal to the distance away from the center. Dots randomly scattered in each direction would indicate no directional bias, whereas an off-center cloud would indicate systematic bias. An angular histogram was also used to explore the direction of displacement. In this angular histogram, frequencies were proportional to the radial length of each slice.

### Statistical analyses

Concordance of area-based characteristics for locations assigned by the two geocoding methods was examined using intraclass correlation coefficients which incorporated information on bias as well as association [24].

Hypotheses regarding differences in discrepancy-distance by area-level characteristics were tested using linear regression models with robust variance estimation in Stata 8.2 [24]. We fit regression models with log-transformed discrepancy-distance as the outcome; predictors were log-transformed population density, log-transformed percent below poverty line, and the most common reference file used for the multi-stage method in that county (three categories). Log-transformations were used to moderate skewness and heteroscedasticity.

We present model results as ratios of geometric means, which are approximately equivalent to ratios of medians. Geometric means or medians are used as measures of central tendency because of the skewed distribution of discrepancy-distances: many discrepancy-distances are quite close to zero, and only a few are above one kilometer, so that the mean is consistently higher than the median (for a normal distribution, the mean and median are approximately equal). A 10% decrease in the median discrepancy-distance can be interpreted as a shift in the distribution such that the midpoint decreases by 10%, even though the skewed shape remains. More generally, this decrease in discrepancy-distances indicates that the two methods are in closer agreement, geocoding the same address to longitudes and latitudes that are closer together.

## Competing interests

The author(s) declare that they have no competing interests.

## Authors' contributions

GSL, RH, EAW, KR, and BMP contributed to the conception, study design, and presentation of the results. GSL and JCW carried out the single-stage geocoding method and comparisons of the geocoded address coordinates from the two geocoding methods. CFE carried out the multi-stage geocoding method and provided descriptions of the multi-stage geocoding process and project addresses. KR provided biostatistical guidance and assisted with the preparation of figures. GSL conducted data analyses and prepared the manuscript. All authors critically reviewed manuscript drafts and approved the final manuscript.
